# 

**Published:** 1891-10

**Authors:** 


ESTABLISHED 1855. SUBSCRIPTION, $2.50.

### THE ATLANTA MEDICAL AND SURGICAL JOURNAL.

EDITED BY

H. V. M. MILLER, M. D., LL. D. L. P. KENNEDY, A. M., M. D.
VIRGIL O. HARDON, M. D. M. B. HUTCHINS, M. D.
F. W. McRAE, M. D. L. B. GRANDY, M. D.

PUBLISHED BY JAS. P. HARRISON & CO., ATLANTA, GA.
Entered at the Post-Office at Atlanta, Ga., as second-class matter.

